# Intraperitoneal infusion of mesenchymal stem cell attenuates severity of collagen antibody induced arthritis

**DOI:** 10.1371/journal.pone.0198740

**Published:** 2018-06-07

**Authors:** Yoojun Nam, Seung Min Jung, Yeri Alice Rim, Hyerin Jung, Kijun Lee, Narae Park, Juryun Kim, Yeonsue Jang, Yong-Beom Park, Sung-Hwan Park, Ji Hyeon Ju

**Affiliations:** 1 CiSTEM Laboratory, Catholic iPSC Research Center, College of Medicine, The Catholic University of Korea, Seoul, Republic of Korea; 2 Division of Rheumatology, Department of Internal Medicine, College of Medicine, The Catholic University of Korea, Seoul, Republic of Korea; 3 Division of Rheumatology, Department of Internal Medicine, Yonsei University College of Medicine, Seoul, Korea; US Department of Veterans Affairs, UNITED STATES

## Abstract

It is unclear how systemic administration of mesenchymal stem cells (MSCs) controls local inflammation. The aim of this study was to evaluate the therapeutic effects of human MSCs on inflammatory arthritis and to identify the underlying mechanisms. Mice with collagen antibody-induced arthritis (CAIA) received two intraperitoneal injections of human bone marrow-derived MSCs. The clinical and histological features of injected CAIA were then compared with those of non-injected mice. The effect of MSCs on induction of regulatory T cells was examined both *in vitro* and *in vivo*. We also examined multiple cytokines secreted by peritoneal mononuclear cells, along with migration of MSCs in the presence of stromal cell-derived factor-1 alpha (SDF-1α) and/or regulated on activation, normal T cell expressed and secreted (RANTES). Sections of CAIA mouse joints and spleen were stained for human anti-nuclear antibodies (ANAs) to confirm migration of injected human MSCs. The results showed that MSCs alleviated the clinical and histological signs of synovitis in CAIA mice. Peritoneal lavage cells from mice treated with MSCs expressed higher levels of SDF-1α and RANTES than those from mice not treated with MSCs. MSC migration was more prevalent in the presence of SDF-1α and/or RANTES. MSCs induced CD4+ T cells to differentiate into regulatory T cells *in vitro*, and expression of FOXP3 mRNA was upregulated in the forepaws of MSC-treated CAIA mice. Synovial and splenic tissues from CAIA mice receiving human MSCs were positive for human ANA, suggesting recruitment of MSCs. Taken together, these results suggest that MSCs migrate into inflamed tissues and directly induce the differentiation of CD4+ T cells into regulatory T cells, which then suppress inflammation. Thus, systemic administration of MSCs may be a therapeutic option for rheumatoid arthritis.

## Introduction

Rheumatoid arthritis (RA) is a systemic autoimmune disease that affects joints and bones. The pathologic hallmarks of RA are synovial inflammation and subsequent destruction of cartilage and bone [1]. In general, the pathogenesis of RA is characterized by overactivation of pro-inflammatory immune responses, resulting in pannus formation and inflamed synovial tissues [2]. However, recent studies suggest that the regulatory function of the immune system in RA patients is impaired. The number of circulating regulatory T (Treg) cells within the peripheral blood mononuclear cell population in RA patients is lower than that in healthy controls and patients with osteoarthritis [3, 4]; also the suppressive capacity of Treg cells in patients with active RA is compromised [5]. The finding that anti-rheumatic drugs such as methotrexate and biologic agents induce a significant rise in both the number and activity of Treg cells [5, 6] also suggests that immune regulatory function plays an important role in the pathogenesis of RA.

Recent advances in RA treatment based on pathophysiological insights have led to a significant improvement in the clinical outcome for patients. However, many patients still fail to reach the therapeutic target under current treatments. Moreover, RA patients often experience adverse events because the therapeutic modalities suppress normal host immune responses. Therefore, there is a need to explore alternative immune modulatory therapies that will restore the balance between the inflammatory and regulatory functions of the immune system. One such strategy is cell-based therapy.

Mesenchymal stem cells (MSCs) are a promising candidate for immune modulatory cell-based therapy. MSCs are adult stromal cells that show increased proliferation and multipotency (i.e., they can differentiate into various cell types). Although MSCs were originally identified as plastic-adherent cells that support hematopoiesis [7], current evidence suggests that they may play a therapeutic role in inflammatory conditions. Intravenous or intraperitoneal injection of MSCs into experimental animal models of inflammatory disease reduces both clinical severity and histologic grade [8]. Moreover, the capacity to proliferate and differentiate makes MSCs an attractive source for cell-based therapy.

The immune regulatory properties of MSCs are thought to be mediated via cell-to-cell interactions. Previous studies suggest that MSCs must make contact with immune cells to induce immune regulatory functions and inhibit pro-inflammatory functions [9–13]. Migration and appropriate recruitment of the injected MSCs are critical for a successful outcome; indeed, several preclinical models report successful treatment of inflammatory disease after local injection of MSCs at inflammatory sites [14, 15]. However, systemic administration of MSCs also alleviates local inflammation, suggesting that injected MSCs migrate to the sites where they are needed [16–20]. Also, the wide range of bioactive molecules (which have immunomodulatory, anti-apoptotic, anti-inflammatory, chemotactic, and regenerative properties) secreted by MSCs have therapeutic efficacy in inflamed lesions [21]. Several studies show that the therapeutic effects of migrated MSCs in various animal models of disease may be due to secretion of chemokines/cytokines. While some notable studies have examined the effects of MSCs in arthritis, none has examined mouse models of collagen antibody-induced arthritis (CAIA).

Here, we examined the therapeutic effects of MSCs in mice with inflammatory arthritis to identify the mechanisms underlying their immune regulatory properties. We compared the clinical and histological severity of CAIA in mice injected with or without MSCs and evaluated local chemokine production and cell migration *in vivo*.

## Materials and methods

### Mice

Six-week-old female DBA1/J mice were purchased from OrientBio (Seongnam, Korea) and maintained under pathogen-free conditions. Arthritis was induced when mice were 7 weeks old. All procedures were undertaken in accordance with the Laboratory Animals Welfare Act and the Guide for the Care and Use of Laboratory Animals, and with the Guidelines and Policies for Rodent Experimentation provided by the Institutional Animal Care and Use Committee of the Catholic University of Korea. The study was approved by the Animal Research Ethics Committee of the Catholic University of Korea (CUMC-2015-0150-01). Animals were housed in groups of five and maintained in an environment-controlled room at 22°C under a 12:12 hour light:dark cycle. To minimize animal suffering, all mice were fully anesthetized under gas anesthesia using isoflurane (2–2.5%) and sacrificed by cervical dislocation.

### Induction of CAIA

Mice were injected intraperitoneally with a cocktail of monoclonal antibodies specific for epitopes on type II collagen (2 mg; Chondrex, WA, USA). After 3 days, mice received 50 μg of lipopolysaccharide (LPS; Chondrex, WA, USA) as previously described [22]. Development of arthritis was monitored every day for 14 days post-injection of the monoclonal antibody cocktail. The severity of arthritis was scored on scale of 0 to 4 in a blinded manner as follows: 0 = normal; 1 = mild swelling confined to the ankle joint or tarsal joints; 2 = mild swelling extending to the mid-foot; 3 = moderate swelling extending to the metatarsal joints; or 4 = severe swelling encompassing the ankle, foot and digits. The scores for all four paws were summed to generate the final arthritis score.

### Preparation and delivery of MSCs

MSCs derived from human bone marrow were purchased from the Catholic Institute of Cell Therapy, South Korea and maintained in Dulbecco’s Modified Eagle’s Medium (DMEM; Gibco, Grand Island, NY, USA) containing 20% fetal bovine serum (FBS; Gibco, Grand Island, NY, USA), 100 U/mL penicillin, and 100 μg/mL streptomycin (Gibco, Grand Island, NY, USA). On the day following injection of LPS, CAIA mice received an intraperitoneal injection of 5 × 10^6^ MSCs in 500 μL of phosphate-buffered saline (PBS), followed by a second injection of 5 × 10^6^ MSCs 3 days later.

### Histopathological analysis

On Day 14 post-injection of anti-type II collagen antibodies, CAIA mice were euthanized and the hind paws fixed in 10% formaldehyde for 2 days. Paws were then decalcified in 10% ethylenediamine tetraacetic acid (EDTA) for 30 days at 4°C and embedded in paraffin. The joint tissues were then cut into 4 μm sections and stained with haematoxylin and eosin (H&E), toluidine blue, and safranin O. The severity of histopathological arthritis was determined on a scale from 0 to 3 as previously described [23]: 0 = no arthritis; 1 = mild arthritis; 2 = moderate arthritis; and 3 = severe arthritis. Synovial inflammation and articular destruction were evaluated according to the extent of cellular infiltration and pannus formation, and loss of cartilage and bone, respectively. Histological scoring was performed by three independent investigators.

### Real-time polymerase chain reaction

Mouse forepaws were homogenized using a gentle MACS Dissociator (Miltenyi Biotec, Bergisch Gladbach, Germany), and samples were incubated with Trizol (Invitrogen, Life Technologies, Carlsbad, CA, USA) to extract mRNA. cDNA was synthesized from 2 μg of extracted total RNA using the RevertAid^TM^ First Strand cDNA Synthesis kit (Thermo Fisher Scientific). Real-time PCR was carried out using a LightCycler® 480 Instrument II (Roche, Basel, Switzerland). Expression of mouse Foxp3 was determined using the following primers: Forward, CAAGGTGAGCGAGTGTCCCTGCTC; and Reverse, AGTTGCCGGGAGCTGGAGTGG. Mean cycle threshold values from triplicate experiments were used to calculate gene expression, which was normalized to gapdh (internal control).

### Isolation of peritoneal cells

The outer layer skin on the abdominal wall was removed to expose the peritoneum covered by the inner layer of skin. Sterile PBS (5 mL) was then injected into the peritoneal cavity using a 5 mL syringe fitted with a 27-gauge needle. After gently rubbing the peritoneum, the peritoneal fluid was collected in the same syringe. The fluid was centrifuged at 1500 × g for 6 min and the supernatant removed. Cytokine and chemokine expression by the isolated cells was then analyzed (see below).

### Mouse cytokine/Chemokine array

A mouse cytokine array was used for simultaneous detection of 62 cytokines according to the manufacturer’s protocol (ab133995, Abcam, Cambridge, MA, USA). Briefly, mouse peritoneal cells were lysed in cell lysis buffer comprising 0.1 M Tris (pH 7.6) containing 0.15 M NaCl and 0.5% Nonidet P-40. The cell lysate was then added to the membrane of a mouse cytokine array. After washing the membrane, the detection antibody was applied and immunoblot images were captured using the BioSpectrum Imaging System. The intensity of each spot was measured using Image J software (version 1.44, NIH, Maryland, USA).

### T cell differentiation and co-culture with MSCs

CD4+ T cells were isolated from CAIA mouse splenocytes using a magnetic sorter and microbeads coated with an anti-CD4 antibody (Miltenyi Biotec, Bergisch Gladbach, Germany). CD4+ T cells were then stimulated with 1 μg/mL plate-bound anti-CD3 (BD Biosciences, San Jose, CA, USA) and 2 μg/mL anti-mouse CD28 (BD Biosciences, San Jose, CA, USA) in RPMI-1640 supplemented with 10% FBS. After 2 h, T cells were differentiated into Treg or type 17 T helper (Th17) cells under specific conditions. Briefly, Treg cells were induced for 3 days in the presence of anti-mouse interleukin (IL)-4 (2 μg/mL), anti-mouse interferon-γ (IFN-γ, 2 μg/mL), and transforming growth factor-β (TGF-β, 1 ng/mL). For Th17 differentiation, CD4+ T cells were treated for 3 days with recombinant IL-6 (20 ng/mL), anti-mouse IL-4 (2 μg/mL), anti-mouse IFN-γ (2 μg/mL), and TGF-β (2 ng/mL). All growth factors were purchased from R&D systems (Minneapolis, MN, USA). To evaluate the *in vitro* effect of MSCs, 5 × 10^4^ MSCs were added to T cell culture on Day 1 of the Treg and Th17 differentiation.

### Flow cytometry

Treg/Th17 cells were cultured in the presence or absence of MSCs and then stained with rat anti-mouse CD4 antibodies conjugated to APC (BD Biosciences, San Jose, CA, USA), and with anti-mouse CD25 antibodies conjugated to APC-Cy7 (BD Biosciences, San Jose, CA, USA). After permeabilizing T cells using a buffer set (eBioscience, Waltham, MA, USA), Treg and Th17 cells were stained with anti-Foxp3 antibodies conjugated to FITC (eBioscience, Waltham, MA, USA), and with anti-human/mouse RORγt antibodies conjugated to PE (eBioscience, Waltham, MA, USA), respectively. Cells were then examined in an LSR Fortessa cell analyzer (BD Biosciences). Data were analyzed using FlowJo 7.6.5 software (TreeStar Inc., Ashland, OR, USA).

### *In vitro* scratch assay

Human MSCs were cultured to 90% confluence in 6-well plates (Corning-Coaster, Tokyo, Japan). The cell monolayer was then scratched with a 200 μL pipette tip to generate a vertical line. MSCs were cultured with PBS/DMEM containing 10% FBS in the presence of 500 ng/mL CXCL12/stromal cell-derived factor-1 alpha (SDF-1α; R&D systems, Minneapolis, MN, USA) and 500 ng/mL CCL5/regulated on activation, normal T cell expressed and secreted (RANTES; R&D systems). MSCs migrating into the wounded area were photographed and counted both before and after treatment with SDF-1α and RANTES. Images were acquired every 2 h between 0 and 12 h. The number of migrating cells was counted by three independent observers.

### Transwell migration assay

Chemotaxis of MSCs was evaluated using commercially available Transwell® polycarbonate membrane cell culture inserts in 24-well plates (CLS3422, Sigma-Aldrich, St. Louis, MO, USA) [24]. The assay system comprised two chambers that were separated by a polycarbonate membrane (6.5 mm in diameter). The membrane is cell permeable, with evenly distributed 8.0 μm pores.

Serum-starved MSCs (1 × 10^4^ cells/250 μL DMEM) were loaded into the upper chamber. The lower chamber was filled with 450 μL of serum-free DMEM containing 0.1% bovine serum albumin (Sigma-Aldrich, St. Louis, MO, USA), 500 ng/mL SDF-1α, or 500 ng/mL RANTES. After 7 h, cells remaining in the upper chamber were removed. Cells migrating to the lower chambers were stained with crystal violet solution and counted by three independent investigators.

### Statistical analysis

Experimental data are expressed as the mean and standard error of the mean (SEM). Statistical significance was determined using Student’s t-test. A p-value <0.05 was considered significant. All data were analyzed using SAS software (v. 9.1; SAS Institute, Cary, NC, USA) and GraphPad Prism software (v. 5.01; GraphPad, San Diego, CA, USA).

## Results

### MSCs alleviate inflammatory arthritis

**Fig 1:**
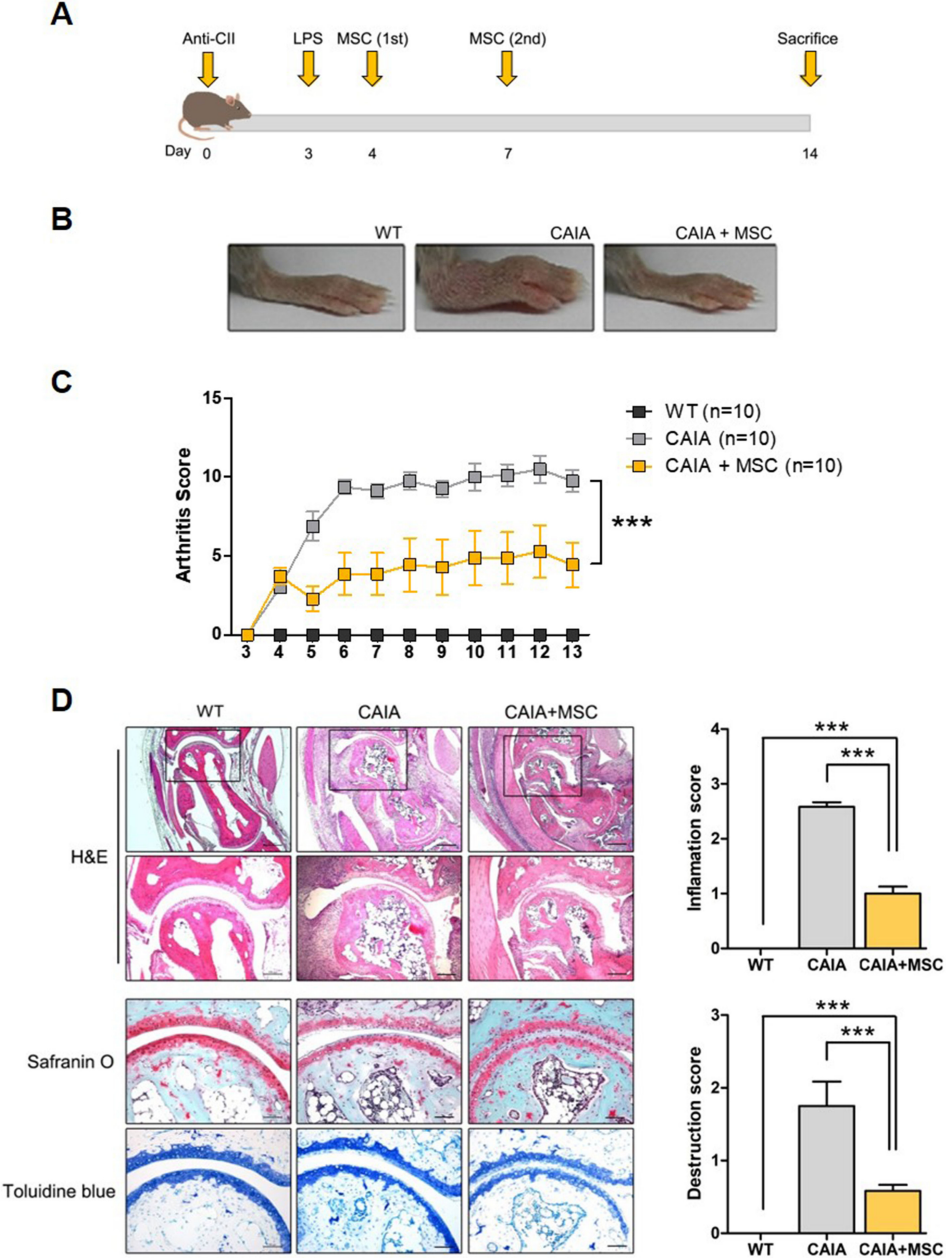
Treatment with MSCs alleviates inflammatory arthritis in mice. (A) Time schedule for induction of CAIA and treatment with MSCs. CAIA mice received two intraperitoneal injections of MSCs (5 × 10^6^ cells each), with a 3-day interval in between. (B) Clinical photographs of the hind paws of wild-type DBA/1J (WT) mice, control CAIA mice (CAIA), and CAIA mice treated with MSCs (CAIA+MSC) at Day 14. (C) Clinical severity of CAIA in each group after injection of a collagen antibody cocktail. Arthritis severity in each paw was scored from 0 (no swelling) to 4 (erythema and severe swelling of entire tarsal joint) and expressed as the mean of the sum of three paws (0–12) ± the standard error of the mean (SEM). (D) Histologic analysis of hind paws from each group at Day 14. Tarsal joints were stained with haematoxylin and eosin (H&E), safranin O, and toluidine blue. Synovial inflammation and bone erosion were evaluated on a scale from 0 to 3. The histological score was expressed as the mean of the scores determined by three independent examiners (± SEM). *** *p* < 0.001.

Mice injected with collagen antibody cocktail developed inflammatory arthritis after LPS administration. CAIA mice received two doses of human MSCs, with a 3-day interval between doses (Fig 1A). Human MSCs significantly attenuated inflammatory arthritis in CAIA mice (Fig 1B). The arthritis score of CAIA mice treated with MSCs did not change significantly after injection with MSCs; however, the score of control CAIA mice increased steadily until it reached a plateau at 6 days post-injection of the collagen antibody cocktail (Fig 1C). The articular structures in MSC-treated CAIA mice were better preserved than those in control CAIA mice (Fig 1D). H&E staining of sections from control CAIA mice revealed prominent infiltration of synovial tissues by inflammatory cells. The results of safranin O staining and toluidine blue staining showed slightly higher levels of cartilage erosion in control CAIA mice than in MSC-injected CAIA mice. Both the inflammation score and the destruction score were significantly lower in CAIA mice treated with MSCs than in control CAIA mice.

### MSCs migrate to inflamed lesions in CAIA mice

**Fig 2:**
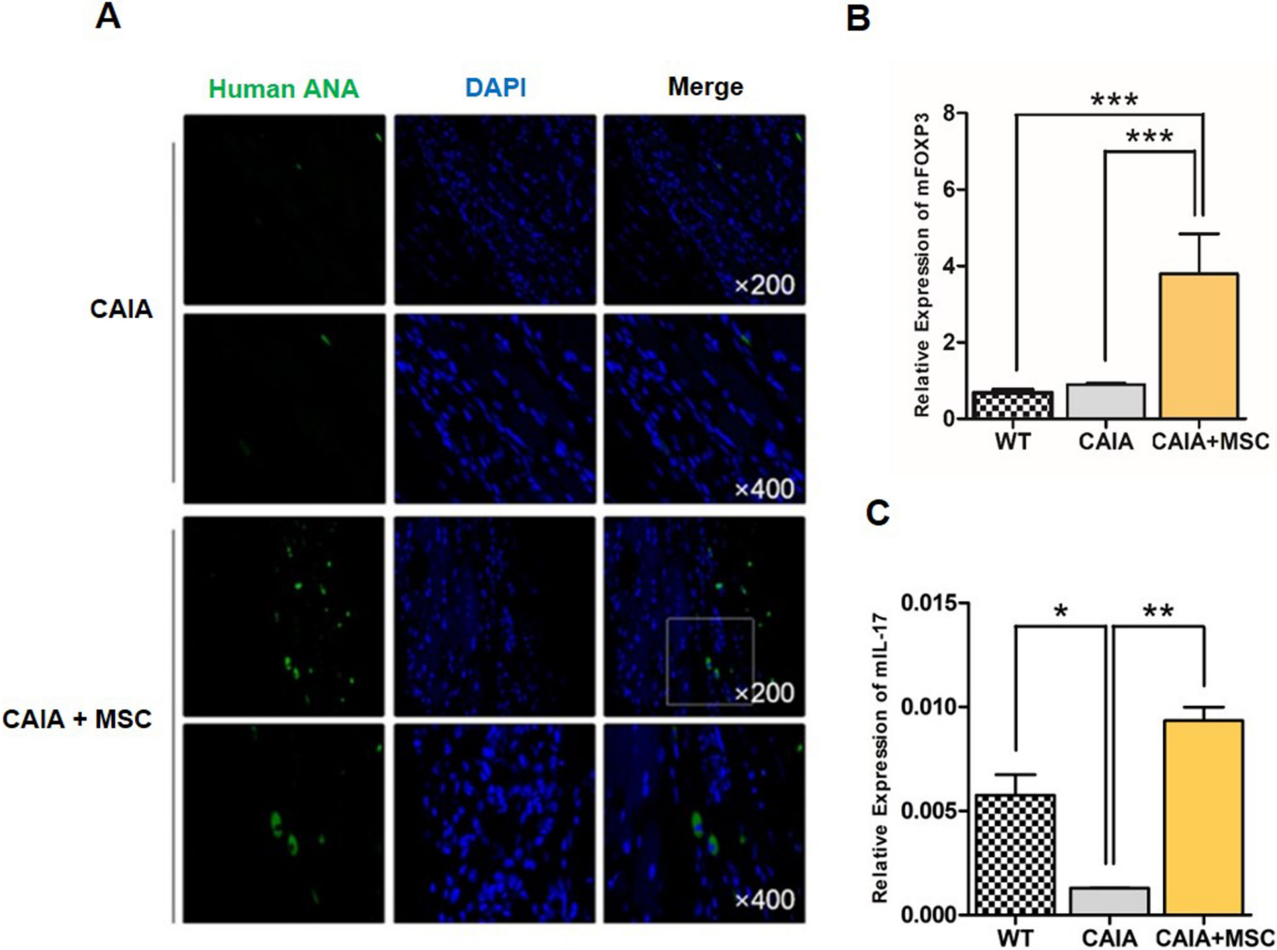
MSCs migrated to inflamed paws and regulated mouse FOXP3 and IL-17 mRNA expression (A) The joints of CAIA mice were stained with human anti-nuclear antibodies (ANAs) to confirm the presence of human MSCs. Expression of (B) mouse FOXP3 mRNA and (C) IL-17 in forepaws of mice from each group. To evaluate *in vivo* expression of mFOXP3 and mIL-17, mRNA was extracted from the homogenized forepaws of CAIA mice from each group. Data are expressed as the mean ± SEM. ** *p* < 0.01; *** *p* < 0.001.

To confirm whether the curative effects were caused by migrated MSCs, we checked for the presence of human cells by staining sections of hindpaws with human anti-nuclear antibodies (ANA). Positive staining for ANA in CAIA mice hindpaws injected with MSCs showed GFP expression, indicating human cells (Fig 2A). No human ANA-positive cells were observed in control CAIA mice. To examine the effects of MSCs *in vivo*, we measured relative expression of FOXP3 and IL-17 mRNA in forepaw homogenates from CAIA mice and MSC-treated CAIA mice. Expression of mouse FOXP3 (mFOXP3) in the forepaw of CAIA mice injected with MSCs increased. Expression was significantly higher (almost eight times) than that in control CAIA mice and wild-type DBA/1J mice (p < 0.001) (Fig 2B). Expression of IL-17 also increased (Fig 2C); however, the change was not as high as that for FOXP3. There were no significant differences between CAIA mice treated with and without MSCs in terms of expression of mFOXP3 or mIL-17 in the spleen (S1 Fig). Previous studies show that MSCs migrate toward inflammatory sites; the data presented herein confirm that MSCs also migrate to inflamed areas in the CAIA mouse model.

### Migrated MSCs promote Treg differentiation *in situ*

**Fig 3:**
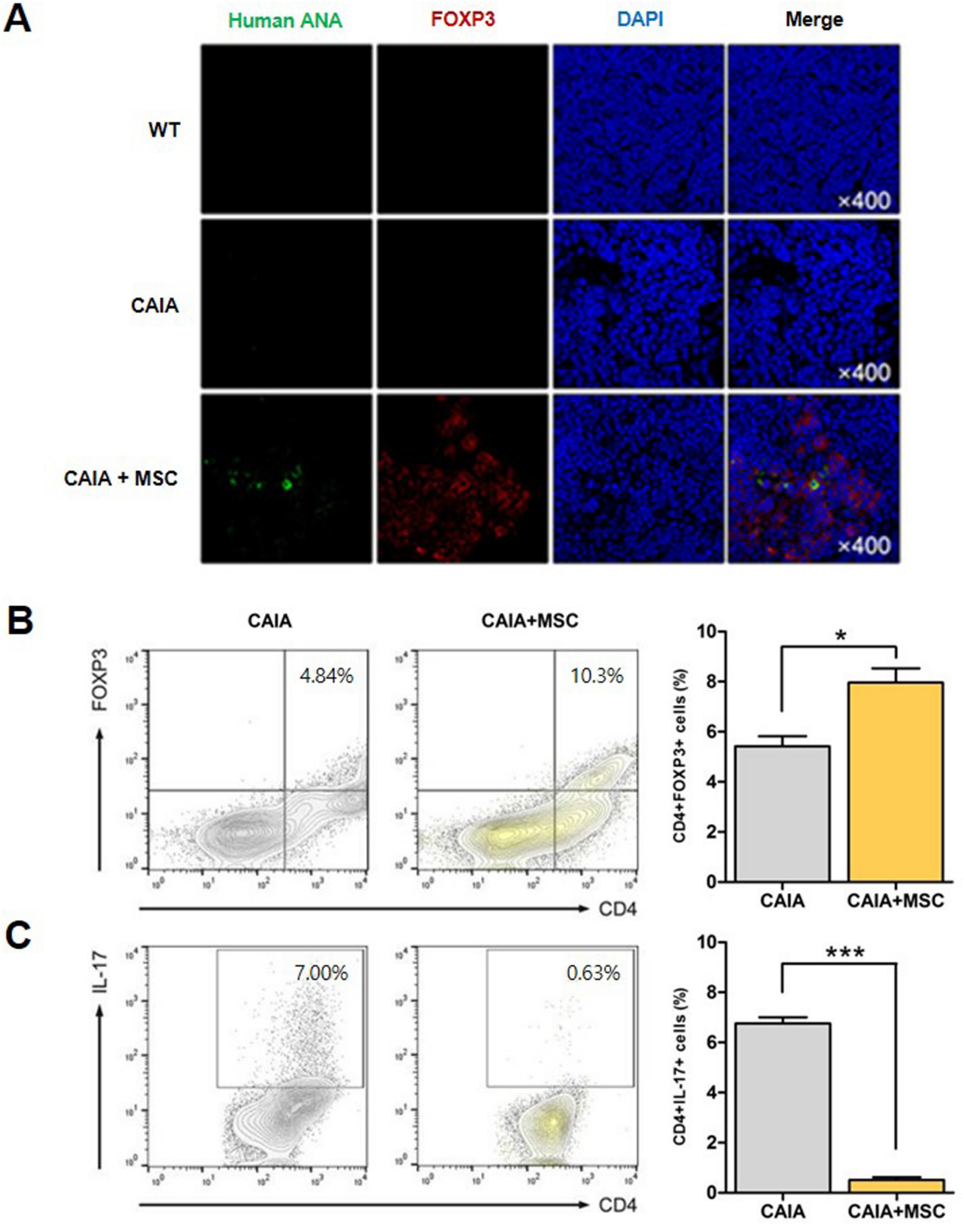
MSCs migrate to the spleen of CAIA mice. (A) Splenic tissues were stained with antibodies specific for mouse FOXP3 to detect FOXP3+ Treg cells. Images of FOXP3+ cells and human ANA+ cells were merged to evaluate the spatial relationship between MSCs and Treg cells. The original magnification is shown in the right panel of each figure. Flow cytometry analysis of CD4+ T cells isolated from splenocytes of control CAIA mice cultured under (B) Treg and (C) Th17 differentiation conditions. Representative images and percentages of each cell types are shown are shown in the graph on the right.

Several reports show that a large proportion of injected MSCs migrate to the spleen [25–28], an organ involved in T cell development and activation. Staining sections of spleen from CAIA mice with human ANAs confirmed the presence of human MSCs among cellular aggregates (Fig 3A). Splenic tissues from CAIA mice treated with human MSCs contained positively-stained cells, indicating migration of injected MSCs. However, no human ANA-positive cells were observed in wild-type mice or control CAIA mice. Spleen sections were also stained with antibodies specific for mouse FOXP3 to assess the loco-regional response by migrated MSCs. Expression of FOXP3 was significantly higher in CAIA mice treated with MSCs than in control CAIA mice. Of note, FOXP3+ cells were predominantly found in close proximity to human MSCs.

CD4+ splenocytes obtained from CAIA mice were cultured with or without MSCs (5 × 10^4^) under conditions that stimulate differentiation into Treg cells or Th17 cells. There was a significant increase in the percentage of CD4+FOXP3+ T cells in the presence of MSCs (4.84% *vs*. 10.3%, respectively; *p* < 0.05) (Fig 3B). MSCs also reduced Th17 differentiation (7.0% (absence) *vs*. 0.63% (presence); *p* < 0.001) (Fig 3C).

### Overexpression of SDF-1α and RANTES induces migration of MSCs

**Fig 4:**
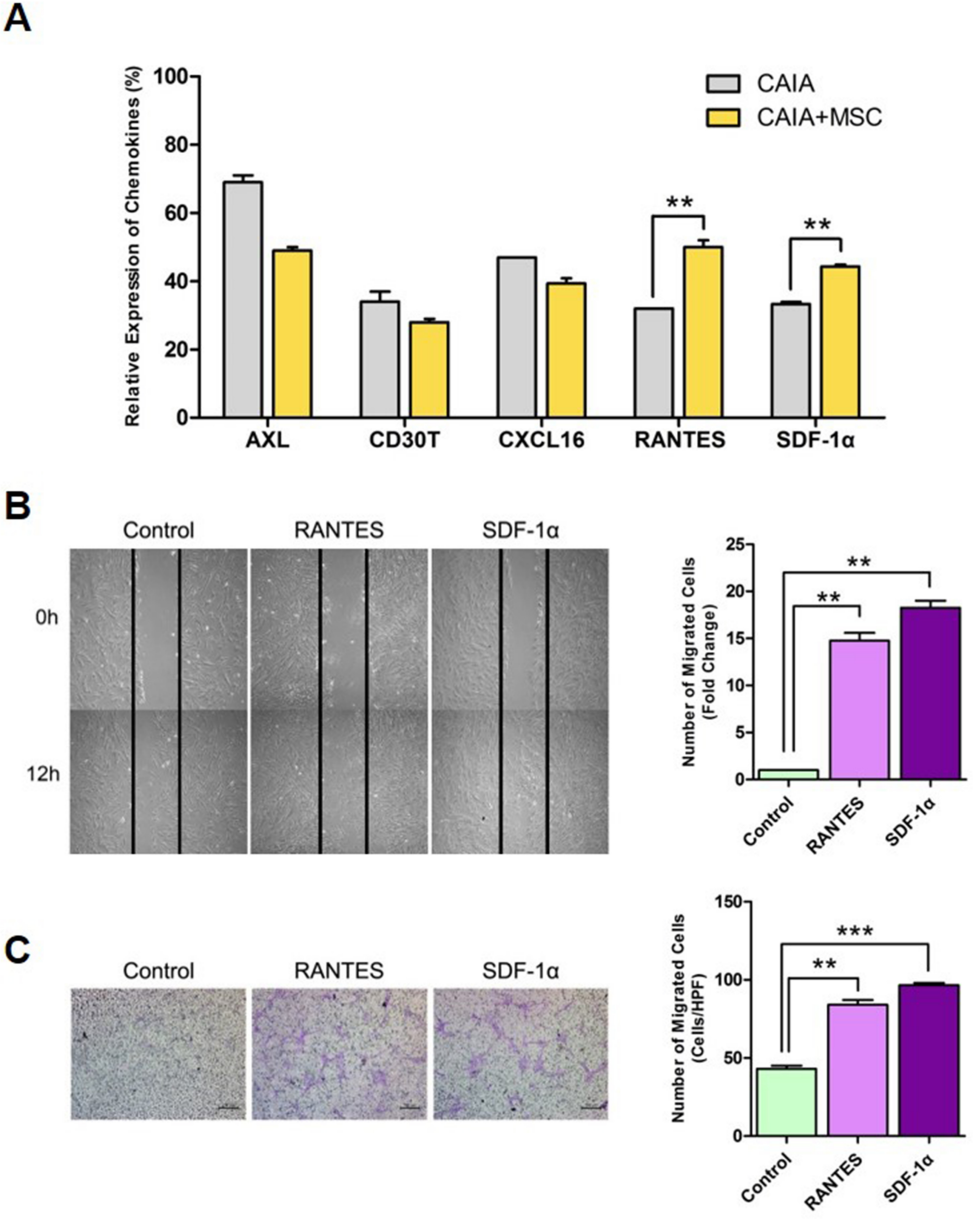
SDF-1α and RANTES induced by MSC treatment promote migration of MSCs. (A) Expression of multiple chemokines in peritoneal mononuclear cells of CAIA mice treated with or without MSCs. Mononuclear cells were isolated from peritoneal wash fluid from CAIA mice. (B) *In vitro* scratch assay to examine MSC migration in the absence or presence of SDF-1α and RANTES. The number of MSCs migrating into the gap was counted every 2 h (between 0 and 12 h) by three independent examiners. Representative images taken at 0 and 12 h are shown. (C) Transwell migration assay to examine MSC migration in response to SDF-1α and RANTES. The two chambers were separated by a cell permeable polycarbonate membrane. The upper chamber was loaded with MSCs and the lower chamber was filled with DMEM containing bovine serum albumin (control), SDF-1α, or RANTES. After 7 h, the number of MSCs migrating into the lower chamber was counted by three independent observers. Data are expressed as the mean ± standard error of the mean (SEM). ** *p* < 0.01; *** *p* < 0.001.

Previous studies suggest that MSCs exert immune regulatory effects by inducing chemokine production. To evaluate regional responses by MSCs, we compared expression of various chemokines in the peritoneal cavity of CAIA mice treated with or without MSCs. Lysates of mononuclear cells isolated from intraperitoneal wash fluid were analyzed using a membrane antibody array (S2 Fig). Among 62 cytokines, signals generated by RANTES and SDF-1α were higher in CAIA mice treated with MSCs than in mice not treated with MSCs (Fig 4A). Because RANTES and SDF-1α promote chemotaxis [29–35], we next examined migration of MSCs in the presence or absence of RANTES and SDF-1α. Migration of MSCs was measured *in vitro* using a scratch assay and a Transwell migration assay. Both assays showed that MSC migration increased in the presence of RANTES or SDF-1α (Fig 4B and 4C). No additive effect was noted. These results suggest that RANTES and SDF-1α may play a role in the curative effects of MSCs in CAIA mice.

## Discussion

The present study shows that treatment of CAIA mice with MSCs alleviates inflammatory arthritis through MSC-mediated induction of Treg cells. Chemokine expression in the peritoneal cavity of CAIA mice treated with MSCs suggests that increased expression of SDF-1α and RANTES promotes migration of MSCs into inflamed joints, where they may induce differentiation of CD4+ T cells into Treg cells.

The immunomodulatory properties of MSCs have been demonstrated in several animal models of inflammatory disease, including collagen-induced arthritis (CIA), experimental autoimmune encephalomyelitis (EAE), and experimental colitis [36]. In line with previous studies, we found that treating arthritic mice with human MSCs improved the clinical and histological grade of arthritis. The CIA mouse model is generated by intradermal injection of type II collagen (emulsified in Complete Freund’s Adjuvant) to induce a pathology that shares many similarities with RA. CIA requires active participation of T and B cells to generate complement fixing antibodies that bind to murine cartilage and initiate a destructive pathology. Toupet and colleagues confirmed migration of intravenously (IV)- or intra-articularly (IA)-injected adipose-derived MSCs in inflamed lesions of CIA mice [37]. T helper 1 cells isolated from spleen or lymph nodes of CIA mice injected IV with adipose stem cells (ASCs) showed decreased populations of CD4+IFN-γ+ cells and CD4+IL17+ cells, but a significant increase in CD4+IL10+ cells. The arthritis score of CIA mice injected with ASCs was also lower than that of CIA mice. In addition, 100% of ASCs injected into wild-type mice and CIA mice were detected in the lung. However, the number of these cells in the lung had fallen by Day 10 post-injection. The authors also confirmed the therapeutic effects in collagenase induced osteoarthritis models. When delivered via IA injection, cells were detected in the muscle and knee joints of mice. More than 90% of injected cells were detected in the knee joint on Day 1; however, cell numbers had fallen by ~30% on Day 10.

Another study by Toupet and colleagues confirmed the effect of IA-injected ASCs in severe combined immunodeficient (SCID) mice [26]. During the early stage post-injection, cells were distributed throughout various organs, including the brain, heart, and blood. However, on Day 186, cells had disappeared from most organs; cells remained only in muscle, fat tissue, and the knee joint. When delivered via IV injection, cells were detected in lung, brain, heart, liver, and joints; however, the number of cells in the knee joint fell by Day 28.

Another study confirmed the effects of MSCs in EAE mice [38]. MSCs were isolated from mouse bone marrow and characterized. In addition, isolated mouse MSCs were co-cultured with T cells and the differentiated cell subtypes analyzed. An MSC-Th1 ratio of 1:10 in co-cultures successfully suppressed differentiation into CD4+IFN-γ+ cells and CD4+IL17A+ cells. Co-culture at a ratio of 1:100 ratio did not lead to a significant increase in inhibition of Th1 differentiation. However, co-culture of MSCs generated CD4+CD25+ cells, and this population tended to increase from Day 0 to Day 4. The increased population of CD4+CD25+ Treg cells was associated with increased levels of IL-10 in the supernatant of co-cultured cells. The reduced population of activated Th17 cells was also caused by increased IL-10 levels.

Previously, we confirmed the therapeutic effects of bone marrow-derived MSCs in interleukin-1 receptor antagonist knock out (IL-1RaKO) osteoarthritis mice [39]. The reduced arthritis score in IL-1RaKO mice was mirrored by a decrease in the thickness of the swollen paw. Intraperitoneally-injected MSCs reduced the CD4+RORγt+ population and increased the CD4+CD25+FOXP3+ population. Expression of several cytokines was also detected in the joints; human IL-1Ra was detected only in mice injected with human MSCs. Expression of mouse IL-1β in MSC-injected IL-1RaKO mice fell, as did that of IL-6, TNF-α and IL-17. Mouse spleen stained positive with an anti-human mitochondria antibody, confirming migration of human MSCs. Mice injected with MSCs expressed human mitochondria; however, the population of cells expressing mouse CD4 fell significantly in regions containing MSCs. When splenocytes from these mice were isolated and Th17 differentiation induced, splenocytes from mice injected with MSCs generated a lower percentage of Th17 cells. Co-culture of MSCs with mouse T cells led to a reduction in Th17 cells, which was caused by human IL-1Ra secreted by human MSCs. Supplementation with human IL-1Ra protein had the same effect on the Th17 population.

Here, we used a mouse model of CAIA. Generation of CAIA bypasses all upstream primary immune responses by triggering anti-type II collagen antibody/complement fixing activity. The downstream (complement cascade/other inflammatory mediators) pathways activated in CAIA models means that the mechanism(s) underlying disease are markedly different from those in different RA animal models; therefore, CAIA mice are a good model for learning more about the mechanisms underlying inflammatory arthritis after LPS stimulation [22]. The major advantage of using CAIA mice for these experiments lies in the fact that there is a short interval between administration of MSCs and development of arthritis. Because histopathological analysis was performed within 7 days of the second MSC injection, we were able to examine their localization in murine tissues by staining sections with human ANAs. Human ANA-positive cells were clearly identified in the inflamed joints and spleen of CAIA mice treated with MSCs, whereas no such cells were detected in CAIA mice not treated with MSCs. In addition, despite the short experimental time, we confirmed that injection of MSCs can alter the T cell population in the spleen and inflamed paw.

We also showed that MSCs exert anti-inflammatory effects by inducing Treg cells. Culturing T cells with MSCs *in vitro* induced Treg cells and suppressed Th17 cells, thereby skewing the Treg/Th17 balance toward Treg cells. Similarly, interaction between MSCs and T cells in the inflamed tissue induced Treg differentiation. Expression of FOXP3 in synovial tissues of MSC-treated CAIA mice was significantly higher than that in untreated CAIA mice, indicating that MSCs induce Treg cells.

Interestingly, MSCs localized to splenic tissue were surrounded by FOXP3+ Treg cells, suggesting that spatial proximity is crucial for MSC-induced T cell differentiation into Treg cells. Previous studies indicate that interaction between MSCs and T cells requires direct cell-to-cell contact, and that paracrine factors have no significant effect [9–12]. Thus, accumulated Treg cells would be expected to originate from the systemic circulation or to be induced *in situ*. The present study suggests that migratory MSCs contribute to induction of Treg cells at inflammatory sites. Although MSCs are thought to have homing properties, few reports show convincing evidence of migration to injured or inflamed sites [40–43]. Most of these studies confirmed the presence of MSCs in local tissues by identifying foreign DNA or chromosomes. Systemically administered MSCs migrate to inflammatory sites and influence T cell differentiation and chemokine production, leading to suppression of local inflammatory responses.

The mechanisms underlying MSC migration involve increased production of chemokines that drive chemotaxis of inflammatory or immune cells. Peritoneal mononuclear cells from CAIA mice treated with MSCs showed upregulated expression of SDF-1α and RANTES. SDF-1α, a ligand of CXC chemokine receptor 4 (CXCR4), is a well-known chemoattractant that induces MSC migration in a dose-dependent manner [29–32]. MSCs express CXCR4 and interact with SDF-1α to increase their homing capacity. Here, we showed that RANTES also promotes MSC chemotaxis. RANTES was originally identified as a paracrine factor involved in chemotaxis of mononuclear cells [33–35]. A single study suggested increased migration of MSCs into the joints of osteoarthritic mice in the presence of RANTES [44]. Thus, we hypothesized that the altered microenvironment induced by MSCs further affects chemotaxis of MSCs, and confirmed that SDF-1α and RANTES promote MSCs migration *in vitro*.

Migration induced by RANTES and SDF-1α is reported by several studies. Ponte and colleagues examined the effect of several growth factors and chemokines on MSC migration [45]. Of nine chemokines selected from the CC, CXC, and CX3C families, only RANTES, MDC, and SDF-1 showed significant chemotactic activity. In addition, when MSCs were treated with TNF-α, they showed increased migration toward RANTES, SDF-1α, and MDC. Further examination confirmed the chemotactic activity of RANTES and SDF-1α by evaluating binding to MSCs. Both RANTES and SDF-1α bound to unstimulated MSCs; however, only RANTES showed increased binding after stimulation with TNF-α. These results are consistent with increased expression of CCR3 (the RANTES receptor), which was only observed in TNF-α-treated MSCs.

Several studies report expression of SDF-1α by MSCs. Liu and colleagues report that SDF-1α protects MSCs from oxidative stress by activating the Akt and Erk survival pathways [46]. In addition, SDF-1α increased secretion of bFGF and VEGF by MSCs. In 2014, Gong and colleagues reported that the SDF-1/CXCR4 axis regulated migration of MSCs toward the pancreas in a rat model of acute pancreatitis [47]. In addition, studies of myocardial ischemia, fracture, and cerebral ischemia report that bone marrow-derived MSCs migrate to sites of inflammation in response to SDF-1α [21, 46, 48–56]. Thus, it is likely that MSC migration in CAIA mice might be driven by RANTES and SDF-1α. Further studies should isolate splenocytes isolated from CAIA mice treated with or without MSCs and treat them with/without RANTES or SDF-1α. In addition, confirming RANTES and SDF-1α expression in tissues of CAIA mice would be useful.

Here, we confirmed that MSCs migrate to the joints of mice with CAIA. Migrated MSCs altered expression of FOXP3 and IL-17 in the mouse forepaw (Fig 2B and 2C). We expected that FOXP3 levels would increase due to induction of immunoregulatory pathways; however, we found that IL-17 levels increased as well. IL-17 levels were lower in control CAIA mice than in wild-type DBA/1J mice or CAIA mice injected with MSCs. IL-17 plays a role in various pathological processes, including inflammation and immune responses; however, studies suggest that it plays a different role after MSC injection [57, 58]. Indeed, one study reports that IL-17 plays a role in MSC migration and motility [59]. However, further studies are required if we are to fully understand the role of IL-17 in CAIA mice and MSCs.

We also confirmed that expression of mFOXP3 was rather unique in the forepaw and spleen (S1 Fig and Fig 2). Although there was no significant difference in FOXP3 mRNA expression in the spleen of CAIA mice treated with or without MSCs, FOXP3 protein levels in CAIA mice injected with MSCs were higher than in those not injected with MSCs. This result is difficult to interpret/explain; however, we assume that it might be caused by a signal that inhibits expression of FOXP3 in the mouse spleen. A previous study shows that FOXP3 is suppressed by several growth factors or cytokines. Huber and colleagues showed that expression of FOXP3 in cultured T cells (induced by TGFβ signaling) was suppressed by IL-27 [60]. Another study confirmed the presence of IL-27 in the spleen and joint tissues of a proteoglycan-induced arthritis (PGIA) model [61]. In addition, another study found increased levels of circulating IL-27 in patients with RA [62]. Therefore, IL-27 may be highly expressed in spleen tissue of CAIA mice; however, no report has examined expression of IL-27 in these mice specifically. Thus, there are several theories that may explain our observations; however, it is clear that further studies are required.

The present study focused on the migratory properties of MSCs and the interaction between MSCs and T cells. To explain the therapeutic mechanism underlying the effects of MSCs on CAIA, regulation of innate immunity by MSCs should be addressed. Previous research indicates that MSCs modulate the behavior of macrophages and dendritic cells, thereby suppressing inflammation. Further studies are required to examine MSC-mediated regulation of innate immune responses that do not involve T cell-mediated mechanisms.

The encouraging results of preclinical studies have led to clinical trials aimed at evaluating the safety and efficacy of MSC-based therapy for autoimmune and inflammatory diseases [63]. Effective delivery of MSCs to sites of inflammation is mandatory for successful cell-mediated therapy. Although the route of MSC administration is still a matter for debate, systemic administration appears to be a feasible approach that results in suppression of local and systemic inflammation. Intravenous infusion of expanded MSC populations into RA patients has shown promising results, with a tolerable safety profile [64]. The homing properties of MSCs would provide a wide range of opportunities to exploit cell-mediated therapy.

Taken together, the results presented herein suggest that MSCs are an effective treatment for inflammatory arthritis because their migration to locally inflamed tissues contributes to immune regulation. Although some issues need to be addressed prior to clinical application, we believe that systemic administration of MSCs could be a feasible therapeutic option for RA patients who are refractory to conventional treatment.

## Supporting information

S1 Fig Expression of mouse FOXP3 and IL-17 in mouse spleen. There was no significant difference between expression levels in wild-type, control CAIA, and MSC-treated CAIA mice.
(JPG)

S2 Fig Image of the chemokine array panel. Expression of Axl, CD30T, CXCL16, SDF-1α, and RANTES increased in the screening panel treated with peritoneal mononuclear cells from CAIA mice treated with MSCs.
(JPG)
