# Supplementary figures and images for: Intraperitoneal infusion of mesenchymal stem cell attenuates severity of collagen antibody induced arthritis
Source: PLoS One. 2018 Jun 7;13(6):e0198740. doi: 10.1371/journal.pone.0198740 (PMC5991665; doi:10.1371/journal.pone.0198740)

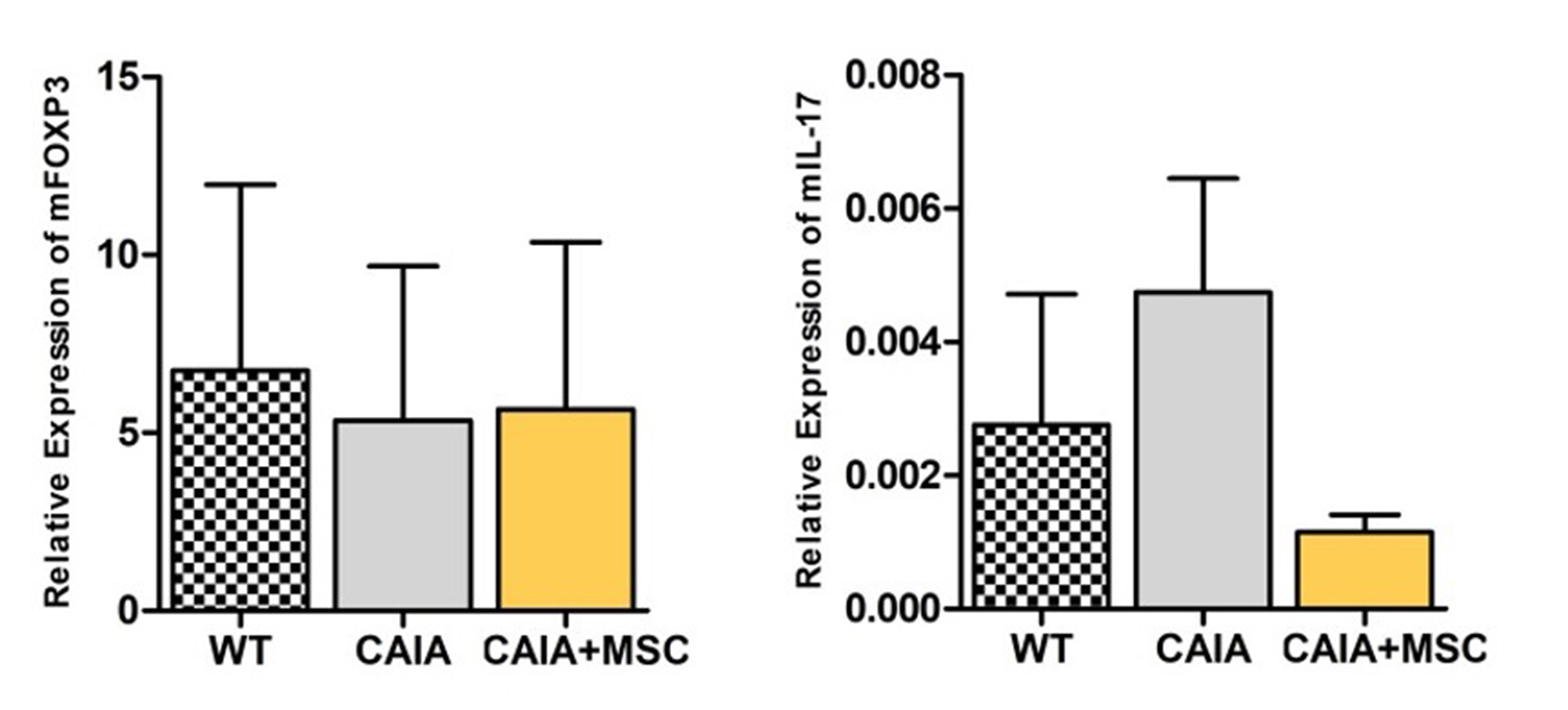

Supplement: S1 Fig — There was no significant difference between expression levels in wild-type, control CAIA, and MSC-treated CAIA mice. (JPG) [file pone.0198740.s001.jpg]

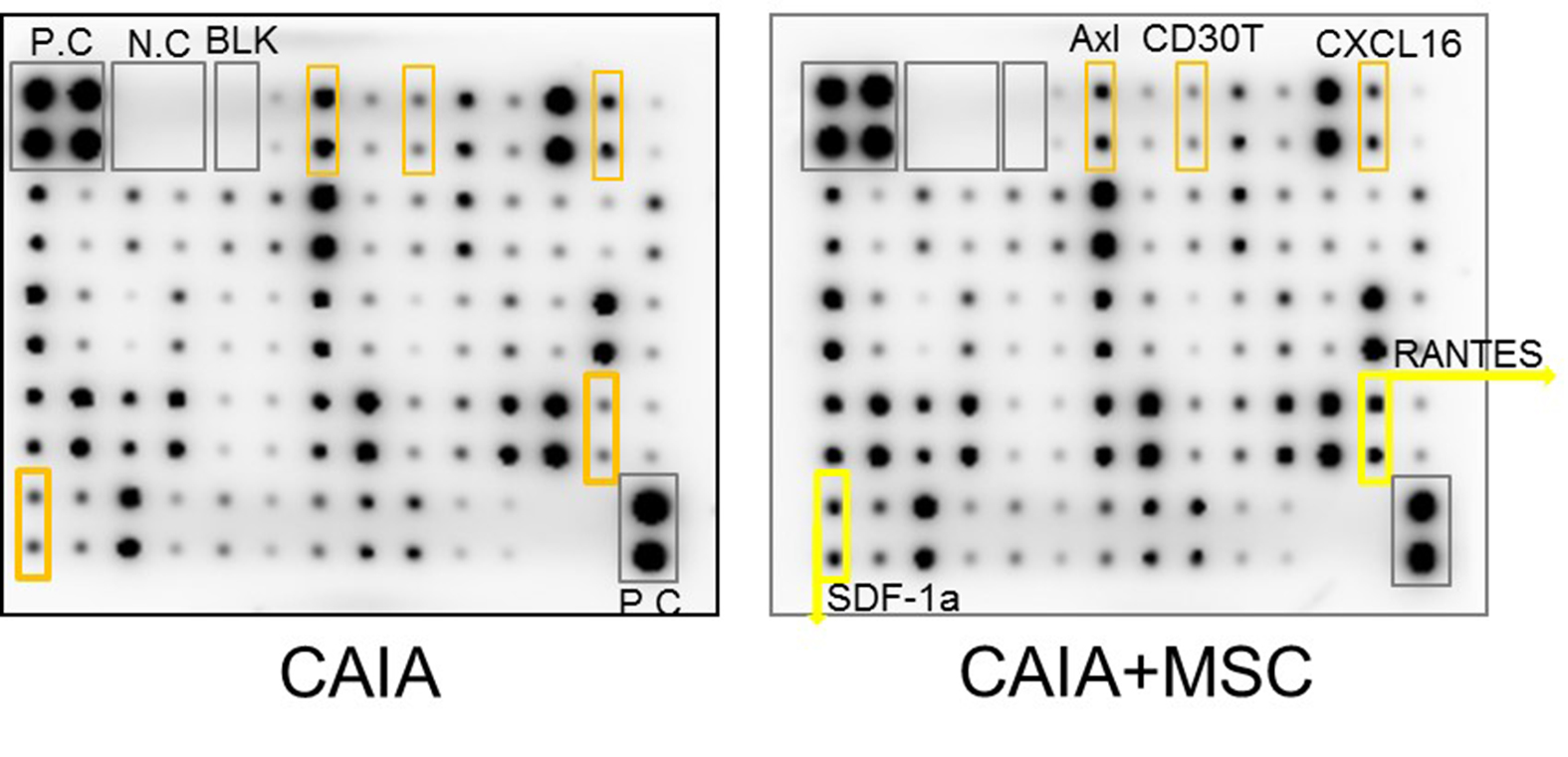

Supplement: S2 Fig — Expression of Axl, CD30T, CXCL16, SDF-1α, and RANTES increased in the screening panel treated with peritoneal mononuclear cells from CAIA mice treated with MSCs. (JPG) [file pone.0198740.s002.jpg]
